# Introductory Lectures

**Published:** 1845-04

**Authors:** 


INTRODUCTORY LECTURES.

The Class of Willoughby University, of Lake Erie, have published the introductory lecture of H. H. Childs, M. D., Prof. of Obstetrics, &c. "General Principles" is the subject discussed in the lecture, and it is treated in a pleasing and masterly manner.

We are much pleased with the Professor's remarks condemning *exclusive* systems, and the distinctions drawn between physicians practising by *rule*, and those who "arrange the *facts* of medicine deducing therefrom a legitimate induction, General Principles, make these the sole guides in his practice." Quackery receives no quarter at the hands of Dr. Childs. Its success he charges in a great measure to the routine practice of the medical profession. We regret that we have not room to quote that portion of the lecture discussing this topic.

---

*Dr. Charles A. Lee*, upon assuming the duties of the chair of General Pathology and Materia Medica, in Geneva College, delivered an admirable lecture upon Medical Education, which has been published by the class. This lecture is far from being an ordinary production. The manner in which the subject is discussed, must prove to the reader the competency of the Professor *to teach*. The lecture, though of unusual length, possesses so much interest, and is written in such an easy and flowing style, that it will be read with pleasure and profit by all who may be so fortunate as to obtain a copy. The reputation which Dr. Lee has made, and is making for himself, as editor and author, would of itself demand for his lecture a careful perusal.

---

An introductory lecture, on the means of promoting the "Intellectual Improvement of the Students and Physicians of the Valley of the Mississippi," by Daniel Drake, M. D., comes to us from the Louisville Medical Institute. No one can be more capable of doing justice to this subject than Dr. Drake. Just returning from a tour through the south and west, with the express view of visiting the medical men of those sections, the opportunities which he has had of observing where improvement is demanded, give the Dr. ample materials for a lecture. An occasional touch of sarcasm (as in his description of a western physician's office,) does not detract from the *interest* of the lecture, and as it comes from a veteran in the profession, will be felt and do good, wherever it strikes home. It would be well for the profession in the Valley of the Mississippi, if every physician and student of medicine were supplied with a copy of Dr. Drake's Lecture.—Ed.

